# Did the Expansion of Insurance Coverage for Oral Health Reduce Self-reported Oral Health Inequalities in Korea? Results of Repeated Cross-Sectional Analysis, 2007–2015

**DOI:** 10.2188/jea.JE20190119

**Published:** 2020-12-05

**Authors:** Nam-Hee Kim, Ichiro Kawachi

**Affiliations:** 1 Department of Dental Hygiene, Wonju College of Medicine, Yonsei University, Wonju, Korea; 2 Department of Social and Behavioral Sciences, Harvard T.H. Chan School of Public Health, Boston, Massachusetts, USA

**Keywords:** health insurance, income, inequality, KNHANES, self-reported oral health

## Abstract

**Background:**

In 2009, the South Korean government expanded universal health insurance to include oral health services. In the present study, we sought to examine whether improved access resulted in a reduction in income-based self-reported oral health inequalities.

**Methods:**

We analyzed repeated cross-sectional data from the Korea National Health and Nutrition Examination Survey (KNHANES) waves IV through VI (2007–2015). We analyzed self-reported oral health status among 68,431 subjects. Changes in oral health inequalities across four income levels (low, middle-low, middle-high, and high) were assessed with the Slope Index of Inequality (SII) and the Relative Index of Inequality (RII).

**Results:**

The average oral health status of children and adolescents improved the most over the observation period. The absolute magnitude of oral health inequalities (measured by the SII) improved for most groups, with the notable exception of young male adults. By contrast, the ratio of poor oral health between high- and low-income groups (measured by the RII) changed little over time, indicating that relative inequalities remained resistant to change.

**Conclusions:**

The expansion of dental health insurance may not be sufficient to move the needle on self-reported oral health inequalities among adults.

## INTRODUCTION

The Republic of Korea achieved universal coverage of health insurance for the population in 1989. However, many challenges remain regarding the performance of the system, including high out-of-pocket (OOP) payments.^1^ Oral health is a critical component of overall health, yet the initial stages of Korea’s health insurance system failed to finance access to oral health care as part of the package. Therefore, the Korean government initiated an expansion of health insurance coverage for dental services in 2009. The expansion sought to improve access to dental care and reduce oral health inequalities. The services covered under the expansion included dental sealants (for caries prevention), dental scaling (for periodontal disease prevention), and fitting of prosthetics (for edentulous rehabilitation).

The main condition for improving oral health inequalities involves providing equitable access to dental care at the national level.^2^^–^^4^ Further work is required to verify whether dental health insurance affects inequalities in oral health beyond access to dental care. Studies conducted in the aftermath of the insurance expansion confirmed that access to dental service was improved^5^^–^^7^; however, disparities in access to services remained.^6^^,^^8^^,^^9^ Currently, few studies have evaluated whether the dental health insurance expansion improved oral health inequalities (not just service access).

There are significant reasons to be cautious in assuming that an expansion in health insurance will achieve a reduction in health inequalities. For example, when the National Health Service was established after the Second World War in Britain, politicians at the time expected it to herald the end of health inequalities. More than 70 years later, however, health inequalities in Britain have not shrunk; instead, they have persisted and in some cases even widened.^10^ Despite universal coverage and dental care reform, oral health inequalities have persisted or even widened in Thailand, Sweden, and Finland.^3^^,^^4^^,^^11^^,^^12^

The continuing inequalities exist because access to health insurance (by itself) is not the *primary* reason for socioeconomic disparities in health status. According to the “social determinants of health” framework, health is determined by multiple “upstream” social circumstances, including the conditions in which people are born, grow, learn, work, and age.^13^ Expanding health insurance can only do so much to redress these social circumstances. Indeed, in some instances, the expansion of access to certain health technologies could even *widen* health inequalities because those who need the services the least (ie, the most advantaged) are better able to take advantage of the expanded services.^14^ The so-called Inverse Care Law—originally formulated by Julian Tudor Hart^15^—maintains that people receive care “in inverse proportion to their level of need”.

Given these debates, we sought to evaluate whether the expansion of health insurance to cover dental services led to a reduction in self-reported oral health inequalities in Korea.

## METHODS

This study design utilized repeated cross-sectional nationally representative data. Our study sample comprised all respondents to the Self-Reported Oral Health (SROH) module (*N* = 68,431; 30,817 men and 37,614 women) of the Korea National Health and Nutrition Examination Survey (KNHANES). Our data were obtained from three waves of the survey (waves IV–VI, corresponding to 2007–09, 2010–12, and 2013–15, respectively). The KNHANES waves are redesigned once every 3 years to every year to provide timely health statistics for monitoring changes in health risk factors and diseases.^16^

We included all age groups (children/adolescents: 0–19 years, young adults: 20–44 years, middle-aged adults: 45–64 years, and older adults: ≥65 years). Sample weights were used to represent the general population.

### Ethical considerations

The KNHANES has been reviewed and approved annually since 2007 by the KCDC Research Ethics. In accordance with ethical guidelines, advance review and approval were obtained from the Institutional Review Board for Human Research, Yonsei University Wonju Severance Christian Hospital (CR318339).

### Variables of interest

Our outcome of interest was self-reported poor oral health, which was assessed via responses to the single item: “How do you rate your oral health, including your teeth and gums?”. The participants chose one of the following responses: “*very good*”, “*good*”, “*average*”, “*bad*”, or “*very bad*”. We combined the “*bad*” and “*very bad*” responses to represent “poor oral health”.

Household income (equivalized for household size) was our indicator of socioeconomic status (SES). We grouped income into four categories by quartiles: low, middle-low, middle-high, and high income. Parents answered instead of children under 12 years of age.

### Calculation of indices of inequality

We calculated two indices of health inequality: the Slope Index of Inequality (SII) and the Relative Index of Inequality (RII). We initially estimated the prevalence of poor oral health (using survey weights) in each wave. The adjusted prevalence and 95% confidence intervals (CIs) for poor oral health according to income rank were calculated. These indices are regression-based measures that consider the whole income distribution, rather than only comparing the two most extreme groups. A ridit score is assigned to each income category based on the midpoint of the range in the cumulative distribution of the population of participants in the given category. Individuals were cumulatively ranked from 0 to 1 according to ascending income position, with “0” representing the lowest income level and “1” representing the highest income. The relative income position variable was then entered as an independent variable in the regression model. SII is the difference in the prevalence of oral health (absolute inequality), and RII is the prevalence rate ratio (relative inequality) between those with the top rank (highest income level) and those with a rank of zero (lowest income level). If there is no inequality, the SII takes a value of zero. Negative SII values indicate a higher prevalence of poor oral health in the low-income group. In general, RII takes positive values; however, RII values can also take on negative values, indicating a gradient in favor of lower-income groups.^17^ The model was adjusted for age and income and stratified by gender. Differences in income inequalities among study years were tested using interaction terms. We pooled the three waves of data from the assessed study years and included an interaction term between SII, RII, and study year for each age group and gender.^18^ All analyses were controlled for age group- and wave-specific fixed effects. We used Stata statistical software (version 15.1) (Stata Corp LP, College Station, TX, USA) for all statistical analyses. R (version 3.5.1, SNU General Public License, Korea; R Foundation for Statistical Computing, Vienna, Austria) was used to visualize the results.

## RESULTS

### Trends in the prevalence of self-reported poor oral health according to age group, gender, and household income

**Figure 1.**
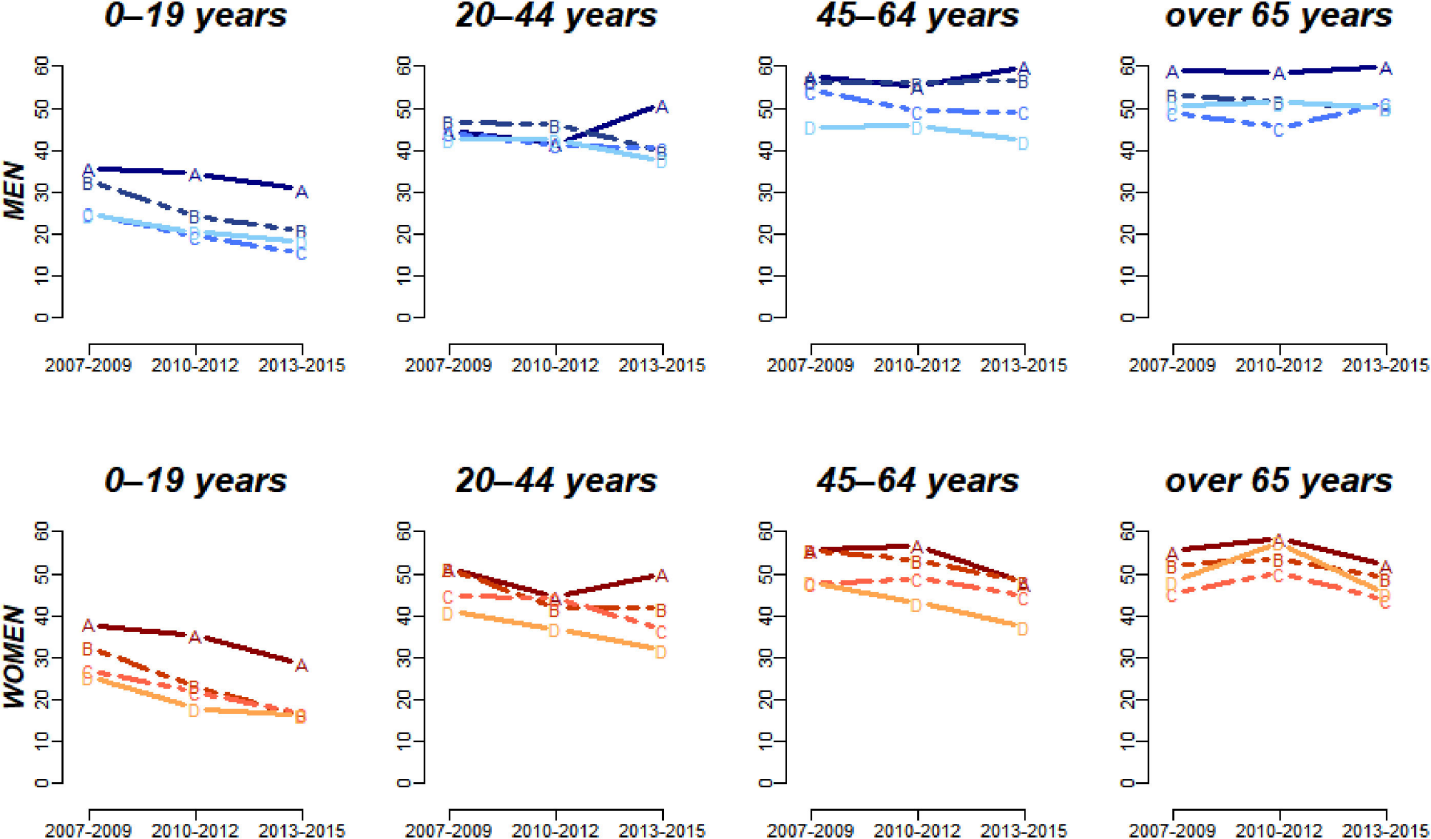
Trends in the prevalence of self-reported poor oral health according to age, gender, and household income. Unit (%) A: Low income, B: Middle-low, C: Middle-high, D: High income.

The age-adjusted prevalence rates of self-reported poor oral health are summarized in Figure 1. Two patterns are evident *within each wave*: first, oral health deteriorates with increasing age; second, there is an income gradient in poor oral health (individuals from lower-income households report worse oral health).

*Across waves*, it is also evident that SROH improved over time for every group (age group, sex, income level). However, there are some exceptions to this overall trend. For example, an improving trend was not observed among men aged 20–44, 45–64, and over 65 years across survey years.

### Trends in oral health inequalities over time

**Figure 2.**
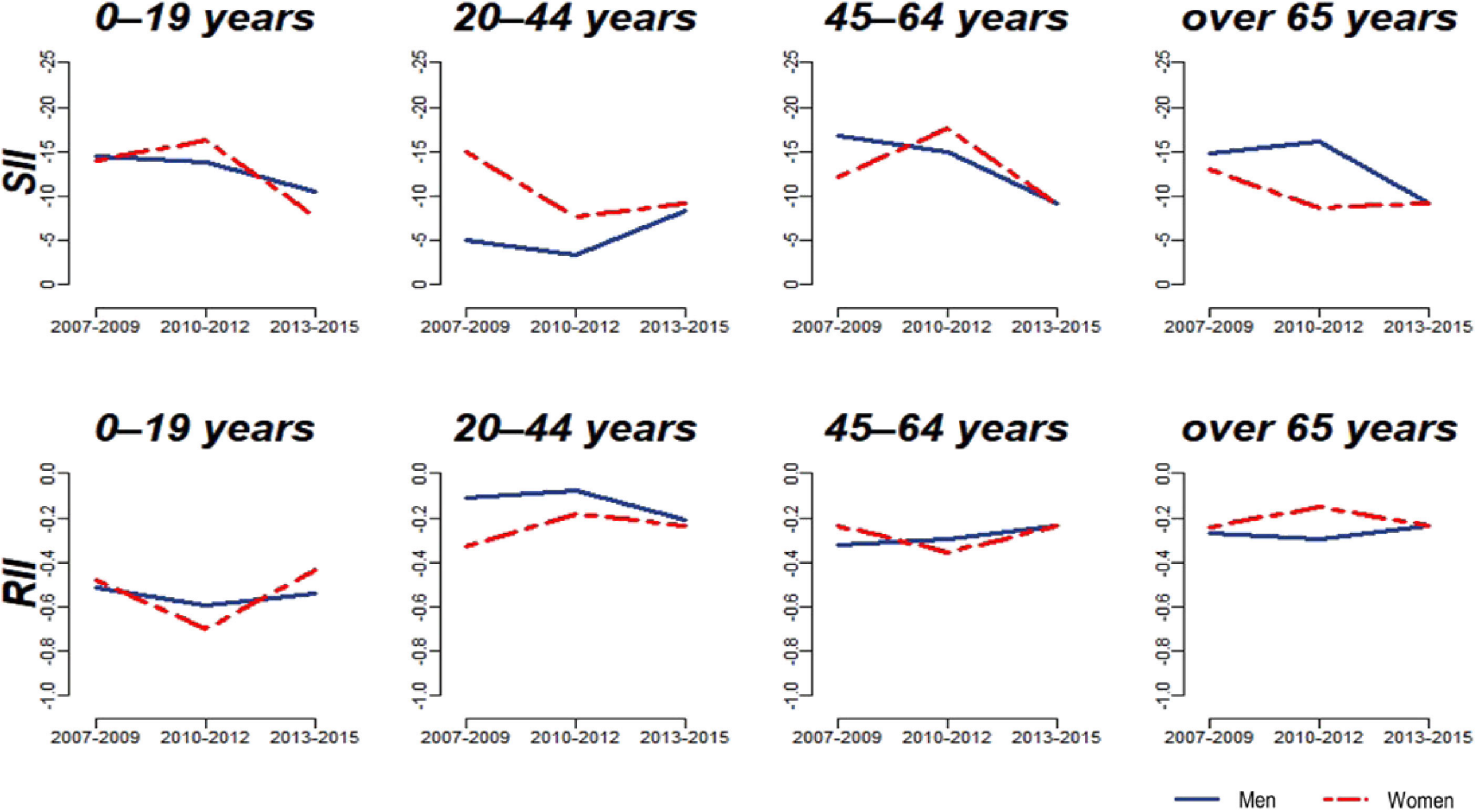
Trends in income-based absolute (SII) and relative (RII) self-reported oral health inequalities over time across age, gender, and study waves

Figure 2 shows the inequality indices of SII and RII. Among children and adolescents, oral health inequalities declined in absolute terms (SII) over the period of observation. This was indicated by the change in SII from −14.4 (2007) to −10.4 (2013) in boys and from −14.0 (2007) to −7.6 (2013) in girls. Conversely, for both boys and girls, oral health inequalities on the relative scale (RII) remained essentially unchanged during the period of observation (RII changed from −0.51 (2007) to −0.54 (2013) in boys and from −0.48 (2007) to −0.43 (2013) in girls). This indicates that, although the absolute size of the gap in poor oral health declined over the 8-year period, the relative inequality (between high- and low-income groups) remained resistant to change (Figure 2).

Similar overall trends were found for all age and sex groups in adults, as demonstrated by a decline in oral health inequalities on the absolute scale, but relatively unchanged inequalities on the relative scale. The one notable exception to these trends was observed among young adult men (Figure 2), among whom inequalities increased on both the absolute and relative scales. The reason for this was that the oral health of high-income males improved, whereas the prevalence of oral health in low-income males deteriorated during the third wave (Figure 1).

## DISCUSSION

The goal of our study was to explore whether the expansion of health insurance (to cover dental services) achieved a reduction in self-reported oral health inequalities by income. Our findings suggest that, although health insurance expansion improved access to dental care and may have contributed to an absolute improvement in self-reported oral health for most groups, the relative income gradient in oral health remained resistant to change.

The reasons for the persistence (or even widening) of inequality after dental care reform have focused on dental care systems that feature a fee-for-service structure, and/or rely on the provision of services from the private sector.^3^^,^^11^^,^^12^^,^^19^ Although the national health insurance in Korea covers the entire population, the proportion of OOP payment in dental services expenditure is high. Several dental services have only just recently been included as benefits of the insurance expansion.

Nevertheless, we observed that oral health inequalities can be reduced over time on the absolute scale, yet they remain unchanged (or even worsen) on the relative scale. The stable or narrowing inequality trends may be related to health changes caused by different factors that affect groups according to their income.^20^ It seems that the decrease in absolute inequality is due to the improvement of oral health among all income groups. The unchanged relative inequality might be due to the fact that self-reported oral health improved more among the better-off than among the poor, potentially due to economic and social changes during this period. This finding has previously been highlighted in other contexts. For example, racial disparities in infant mortality in the United States have improved over time on the absolute scale, with both black and white infants experiencing an absolute decline in mortality.^21^ This has also resulted in a lower SII over time. However, the *relative* gap between blacks and whites has remained very persistent (and even increased).^22^^,^^23^

In the field of oral health, an age-period-cohort analysis in the United Kingdom (spanning the period from 1988 to 2009) showed that in the entire adult population, although absolute inequalities in tooth loss narrowed over time, relative inequalities increased steadily.^24^ It is a matter of debate as to whether the persistence of relative inequalities represents a problem for public health. On the one hand, the “widening” of relative inequalities can be the product of a mathematical artifact: it is easier to generate large ratio measures with falling rates of bad health. On the other hand, the presence of between-group inequalities can be viewed as an indicator of persistent injustice.

In the United States, Medicaid coverage of dental services has increased access to dental care (by reducing OOP costs paid by beneficiaries under the Affordable Care Act)^25^ and may have contributed to an improvement in oral health status. However, improved access to dental care may not be sufficient to close the gap in oral health status because oral health is determined by many other factors outside of health services (eg, water fluoridation and the use of fluoridated toothpaste).

An encouraging finding from our analysis is that income-based *absolute* inequalities in self-reported oral health improved for most groups. Children/adolescents recorded the best improvements in oral health status in addition to reduced inequalities (Figure 1 and Figure 2). The treatment of dental fissures was included for the first time in the health insurance coverage in 2009. The insurance covered just the first molars of 6- to 14-year-olds. Even so, a sharp decline in dental caries was recorded among 8- to 12-year-olds in Korea between 2006 and 2012.^26^ In 2013, the insurance was expanded again to cover the second molars and all age groups under 18 years old.

Despite these achievements, we also noted some remaining areas of concern. Young males bucked the overall trend in terms of widening inequalities; self-reported oral health in women improved more than that in men (even though the insurance expansion covered everyone), and that in the worst off groups did not improve the most. The inequalities appeared to be related to the time since the last dental care visit and income itself in this age group.^12^ Young males tend to have greater and more severe dental caries and periodontal disease compared to women, but they are less likely to have received dental care.^27^ The reasons seem related to their low level of oral health perception or concern.^28^^,^^29^ Men did not appear to use dental services more often after the health insurance expansion.^6^

Annual dental scaling has been covered by insurance since 2013 for everyone aged ≥20 years. Although dental scaling visits remain more common among high socioeconomic groups,^6^ all outpatient dental visits have been reported to increase among middle-aged Koreans after the insurance expansion.^5^ We verified that dental service utilization increased as a result of reduced OOP expenses.^7^^,^^25^^,^^30^ Whether these results are linked to oral health inequalities requires confirmation over a longer period of time.

Since 2012, denture treatment has been offered for people aged ≥75 years every 7 years. The coverage was expanded to everyone ≥70 years in 2015 and again for everyone ≥65 years in 2016. The patient cost amounts to 10–30% of the total fee depending on the income level of the patient and treatment materials. Many edentulous older adults may nonetheless believe (erroneously) that once all their teeth have been extracted, they no longer need to be concerned about oral health.^31^ This may be one of the reasons why the high prevalence of poor oral health among older adult men has not changed. Our results of inequality among older adults might be due to cohort-related historic inequality-shaping forces and the high costs associated with dental care.^4^

In previous studies, SROH has shown a high reliability with clinical oral health outcomes.^32^ Thus, SROH is regarded as a practical and easily interpreted measure to assess inequalities in the oral health of a population.^11^^,^^12^^,^^33^^,^^34^ We confirmed the same patterns of the outcome variable through a sensitivity analysis and robustness check. The supplemental table (eTable 1) shows the results separating “poor” versus “very poor”. However, we found that the proportion of “very poor” self-reported oral health status increased among children/adolescents in the low income group during the survey years from 2013–2015. This indicates a sub-group of special concern, given the two-fold increase, and the likely persistence of poor oral health status as the children advance toward adulthood.

One strength of our research is that we examined all age ranges from children to older adults. We used KNHANES data, so our results can be generalized to the entire population in South Korea. In addition, we could identify population-level trends from the long-term repeated cross-sectional data. Nevertheless, we also note several limitations. First, we cannot draw inferences about the impact of dental insurance expansion on self-reported oral health inequalities because the trends in self-reported oral health that we found appear to have already started *before* the health insurance expansion. Because the Korean health insurance expansion was *national* in scope (as opposed to varying by state, as in the Affordable Care Act in the United States),^35^ we lack a “control” group to be able to identify any causal effects of the policy change (eg, through difference-in-difference estimations).

Second, we have considered only income-based health inequalities. Current household income may not adequately represent the standard of living for different generations nor the cumulative socioeconomic position of households across the life course.^36^ Although the main outcome was self-reported poor oral health, it may be subject to bias due to under- or over-reporting by different groups.

In conclusion, the expansion of dental health may have contributed to an absolute improvement in self-reported oral health for most groups, but the relative income gradient remained resistant to change. Thus, the expansion of dental health insurance may not be sufficient to move the needle on self-reported oral health inequalities among adults so far. Future trends in self-reported oral health inequalities must be monitored with long-term data.

## APPENDIX A. SUPPLEMENTARY DATA

eTable 1. Stratified prevalence of self-reported poor and very poor oral health according to income by age, gender, and survey years
