# Supplementary material for: Did the Expansion of Insurance Coverage for Oral Health Reduce Self-reported Oral Health Inequalities in Korea? Results of Repeated Cross-Sectional Analysis, 2007–2015
Source: J Epidemiol. 2020 Dec 5;30(12):537–41. doi: 10.2188/jea.JE20190119 (PMC7661332; doi:10.2188/jea.JE20190119)
Supplement: Supplementary file 1 [file je-30-537-s001.pdf]

eTable 1. Stratified prevalence of self-reported poor and very poor oral health according to income by age, gender, and survey years

Category	Men						Women					
	K-IV (2007–2009)		K-V (2010–2012)		K-VI (2013–2015)		K-IV (2007–2009)		K-V (2010–2012)		K-VI (2013–2015)	
	Poor	Very poor	Poor	Very poor	Poor	Very poor	Poor	Very poor	Poor	Very poor	Poor	Very poor
**Childhood/adolescence**												
Low	30.3	5.4	32.8	1.6	28.2	2.4	35.0	2.9	31.6	3.5	21.1	7.4
Middle-low	27.7	4.9	22.3	2.0	17.7	3.1	31.2	1.1	20.4	2.7	14.0	2.0
Middle-high	22.3	2.4	18.0	1.2	14.6	1.0	25.4	1.3	20.0	1.7	14.7	1.8
High	23.3	1.2	19.2	1.2	16.9	1.3	23.2	2.0	17.0	0.8	16.1	0.1
**Young adults**												
Low	39.6	4.9	33.0	8.5	42.4	8.5	45.2	6.0	37.2	7.1	42.8	7.2
Middle-low	38.2	8.5	37.8	8.2	31.4	8.3	41.7	9.5	35.7	6.2	33.8	8.1
Middle-high	36.6	7.6	36.4	4.8	34.4	6.1	38.4	6.4	39.6	4.5	31.8	4.8
High	36.6	5.9	37.6	5.0	31.7	5.8	36.5	4.3	32.4	4.3	28.0	3.6
**Middle-old adults**												
Low	45.4	12.3	37.3	17.9	46.2	13.7	45.5	10.2	44.1	12.7	37.5	10.2
Middle-low	44.6	11.7	41.8	14.2	39.5	17.2	46.1	9.4	39.7	13.4	37.0	11.0
Middle-high	43.5	10.8	37.7	11.7	37.0	12.1	39.7	8.1	41.0	7.9	36.7	7.9
High	38.1	7.5	35.9	9.8	32.5	9.6	42.5	5.7	33.6	9.3	29.6	7.7
**Older adults**												
Low	49.7	9.4	47.1	11.5	45.8	14.1	49.1	6.2	47.5	11.0	42.5	9.8
Middle-low	45.4	7.9	43.1	8.5	38.1	12.1	46.8	5.3	45.2	8.3	41.1	7.7
Middle-high	43.8	5.0	37.0	8.3	39.8	11.3	41.4	3.8	43.2	7.0	36.4	7.1
High	43.5	7.0	35.1	16.3	39.2	10.6	43.8	4.3	47.2	10.3	40.1	5.0

Values reported as percentages.
